# Prediction of outcome in patients with non-small cell lung cancer treated with second line PD-1/PDL-1 inhibitors based on clinical parameters: Results from a prospective, single institution study

**DOI:** 10.1371/journal.pone.0252537

**Published:** 2021-06-01

**Authors:** Konstantinos Rounis, Dimitrios Makrakis, Chara Papadaki, Alexia Monastirioti, Lambros Vamvakas, Konstantinos Kalbakis, Krystallia Gourlia, Iordanis Xanthopoulos, Ioannis Tsamardinos, Dimitrios Mavroudis, Sofia Agelaki

**Affiliations:** 1 Department of Medical Oncology, University General Hospital, Heraklion, Crete, Greece; 2 Division of Oncology, University of Washington Medical School, Seattle, Washington, United States of America; 3 Laboratory of Translational Oncology, School of Medicine, University of Crete, Heraklion, Crete, Greece; 4 Department of Computer Science, University of Crete, Heraklion, Crete, Greece; Nine of July University (UNINOVE): Discipline of Medical Oncology - Post Graduation Program in Medicine, BRAZIL

## Abstract

**Objective:**

We prospectively recorded clinical and laboratory parameters from patients with metastatic non-small cell lung cancer (NSCLC) treated with 2nd line PD-1/PD-L1 inhibitors in order to address their effect on treatment outcomes.

**Materials and methods:**

Clinicopathological information (age, performance status, smoking, body mass index, histology, organs with metastases), use and duration of proton pump inhibitors, steroids and antibiotics (ATB) and laboratory values [neutrophil/lymphocyte ratio, LDH, albumin] were prospectively collected. Steroid administration was defined as the use of > 10 mg prednisone equivalent for ≥ 10 days. Prolonged ATB administration was defined as ATB ≥ 14 days 30 days before or within the first 3 months of treatment. JADBio, a machine learning pipeline was applied for further multivariate analysis.

**Results:**

Data from 66 pts with non-oncogenic driven metastatic NSCLC were analyzed; 15.2% experienced partial response (PR), 34.8% stable disease (SD) and 50% progressive disease (PD). Median overall survival (OS) was 6.77 months. ATB administration did not affect patient OS [HR = 1.35 (CI: 0.761–2.406, p = 0.304)], however, prolonged ATBs [HR = 2.95 (CI: 1.62–5.36, p = 0.0001)] and the presence of bone metastases [HR = 1.89 (CI: 1.02–3.51, p = 0.049)] independently predicted for shorter survival. Prolonged ATB administration, bone metastases, liver metastases and BMI < 25 kg/m^2^ were selected by JADbio as the important features that were associated with increased probability of developing disease progression as response to treatment. The resulting algorithm that was created was able to predict the probability of disease stabilization (PR or SD) in a single individual with an AUC = 0.806 [95% CI:0.714–0.889].

**Conclusions:**

Our results demonstrate an adverse effect of prolonged ATBs on response and survival and underscore their importance along with the presence of bone metastases, liver metastases and low BMI in the individual prediction of outcomes in patients treated with immunotherapy.

## Introduction

Immune checkpoint inhibitors (ICIs) targeting the PD-1/PD-L1 axis have demonstrated substantial clinical activity in metastatic NSCLC and received regulatory approval for use as first or subsequent lines of therapy [1–5]. However, only a small proportion of individuals will experience durable clinical remissions and subsequent significant clinical benefit. In addition, beyond PD-L1 levels in tumor cells or the immune cells of the tumor microenvironment, there is currently a lack of biomarkers for the prediction of treatment outcomes. From the financial perspective, the large scale use of these inhibitors is associated with substantial expenditures for the healthcare system, thus rendering their cost-effectiveness debatable [6, 7].

Pretreatment weight loss and low body mass index values have been well-recognized adverse prognostic features in cancer patients [8]. Furthermore, several clinical studies have reported the prognostic value of systemic inflammation in malignancy and the role of routine blood parameters as potential inflammatory biomarkers [9]. Neutrophil/lymphocyte ratio (NLR) and low albumin levels have been associated with treatment outcomes in patients with advanced cancer, including lung cancer [10, 11]. Evidence is being progressively gathered on the application of the aforementioned parameters for the creation of predictive models in a wide spectrum of malignancies [12–14].

Intestinal microbiome composition exerts a pivotal impact in the shaping of an effective immune response [15]. Preclinical data have highlighted the importance of gut microbiota on immunotherapy efficacy in experimental mouse melanoma models [16]. More importantly, antibiotic (ATB) administration may significantly alter the microbiome composition leading to gut dysbiosis and immune dysfunction [17]. Beyond ATBs, proton pump inhibitors (PPis) are among the most common prescribed drugs worldwide and their administration has been linked with a significant decrease in Shannon’s diversity accompanied with alterations at the range of 20% of the bacterial taxa of the intestinal flora [18].

Daily steroid requirements > 10 mg of prednisone equivalent consisted an exclusion criterion for the registrational trials of ICIs [1–5]. In retrospective studies steroid administration has been associated with poor outcomes in patients with NSCLC treated with ICIs [19]. However, besides per os or intravenous steroids, NSCLC patients commonly use inhalational steroids due to the high prevalence of chronic obstructive pulmonary disease (COPD) in these individuals. Inhalational steroids exert a plethora of immunomodulatory effects on bronchial mucosa [20] however their effect on ICI efficacy has not been investigated so far.

Based on the above data we assumed that routinely available clinical and laboratory parameters may have prognostic and predictive relevance in patients with advanced NSCLC treated with ICIs. To test our hypothesis we conducted a prospective observational study in order to evaluate their role in the determination of clinical outcome in patients with metastatic NSCLC treated with ICIs in the second line treatment setting. In addition, we introduced these parameters in the Just Add Data Bio (JADBio) (www.jadbio.com) machine learning pipeline [21, 22] for further multivariate analysis in order to estimate their integrative predictive value in NSCLC.

## Materials and methods

### Study design

This is a prospective observational study enrolling patients with metastatic NSCLC, without EGFR mutations or ALK translocations, treated with ICIs following progression on previous platinum-based chemotherapy. Patients were recruited at the Department of Medical Oncology, University General Hospital of Heraklion, from November 15, 2017 until November 21, 2019. Patients were eligible if they received ICIs as second-line treatment as per standard treatment guidelines, according to the decision of the treating physician. Written informed consent was obtained from all patients before enrollment. The study was reviewed and approved by the institutional review board of the University Hospital of Heraklion and was conducted in accordance with the principles of the Declaration of Helsinki (ID 2644).

### Data collection and outcome assessment

Patients with *EGFR* mutations or *ALK* translocations were excluded from the analysis. Radiological assessment was prospectively performed using CT scans (or MRI if clinically indicated) from the start of immunotherapy and every 8–9 weeks thereafter. Partial response (PR), stable disease (SD) and progressive disease (PD) were assessed according to RECIST 1.1 criteria [23]. Disease stabilization (DS) was defined as the achievement of PR or SD after ICI administration. Disease progression was defined as radiological progression or death during the course of treatment. Progression free survival (PFS) was defined as the time duration between the initiation of immunotherapy and disease progression or death. Overall survival (OS) was defined as the time duration between the initiation of immunotherapy and death. Individuals that had not progressed or were alive at the time of data analysis were censored for PFS and OS respectively at the date of last follow up.

Data on patient [age, gender, smoking status, performance status (PS), body mass index (BMI)], disease characteristics (histology, organs affected with metastatic disease) and context and duration of co-medications [per os (pos), intravenous (iv) or inhalational steroids, ATB and PPis] were prospectively collected. Disease burden was classified as high and low (> 2 and ≤ 2 organs with metastases) at the beginning of immunotherapy. Patients were classified based on their BMI at the start of immunotherapy in a binary fashion with the value of 25 kg/m^2^ used as the cut-off to define BMI high vs BMI low. Common laboratory parameters such as baseline lactate dehydrogenase (LDH) levels, albumin and absolute white blood cell counts were collected at the time of treatment initiation. Elevated LDH levels were defined according to the upper limit of normal value range (UNL) (247 units/liter) and the cut-off for neutrophil/lymphocyte ratio (NLR) was set at > 3. The cut-off for albumin levels was set at 3.5 g/dl that represents the lower normal limit. PD-L1 assessment, when available, further categorized the patients as PD-L1 positive or negative. PD-L1 expression was scored as % of tumor cells showing membranous and/or cytoplasmic staining; the cut-off for positivity was set at ≥ 1%.

Patients were categorized as having received steroids per os or iv in case of steroid use at a dosage of > 10 mg prednisone equivalent for ≥ 10 days within the first 12 weeks of treatment or within 15 days before its initiation. Patients were further sub-classified into two different subgroups, those who had received steroids due to immune related adverse effects (irAEs) and those who had received steroids for supportive reasons (e.g. brain edema due to brain metastases, anorexia, dyspnea, COPD exacerbations). We categorized patients as having been administered ATB if they had received ATBs within 30 days before the initiation of immunotherapy and/or within the first 12 weeks of treatment; prolonged ATB administration was defined as ATBs use for ≥ 14 days. In case of multiple courses of shorter periods, the total duration was calculated. Long-term PPis usage was defined as the use of PPis for a time duration ≥ 3 months before the initiation of immunotherapy. Chronic administration of inhalational steroids was defined as use for ≥ 3 months prior to the start of immunotherapy. The cut-offs of 10 days, 14 days and 3 months for steroids, ATBs and PPIs use, respectively, were set arbitrarily before the initiation of data collection.

### Statistical analysis

Statistical analysis was performed using SPSS 25.00. Descriptive statistics were performed to define categorical and continuous nominal variables. Statistical significance was set at p < 0.05 (two-sided test). Chi square test was used to access any potential associations between each variable with PR and DS rates. In addition, chi-square test was applied to investigate any potential associations of various clinical characteristics with prolonged ATB administration. Mann-Whitney U test was applied to test the effect of duration of ATB administration in days as a continuous variable on DS rates. In addition, we performed binary logistic regression analysis in order to examine the odds ratios (OR) of the studied covariates on the probability of achieving DS as response to ICI administration.

The Kaplan Meier method was used to access any effect of the studied parameters on PFS and OS. Curves were compared with the log-rank test. We initially applied Cox Regression Method to examine the effect of the duration of ATB administration as a continuous nominal variable in days on PFS and OS. Finally, we conducted a univariate analysis for each studied categorical variable and afterwards a multivariate analysis including the parameters that had reached statistical significance in the univariate analysis using Cox Regression Method to investigate their effect on survival outcomes.

We did not perform a sample size and power calculation because at the time of the initiation of data collection there was a scarcity of published reports on the effect of the studied parameters on the outcome of immunotherapy treated cancer patients. Thus, it would have been of no value in this exploratory study due to the lack of available data on which to base the required calculations.

### Multivariate analysis by JADBio tool

For the purpose of conducting a multivariate analysis on our data, we applied JADBio, a fully automated machine learning (AutoML) system (www.jadbio.com). JADBio selects the algorithms and methods corresponding to the particular problem, according to the type of data used and possible preferences set by the user. To do this, it employs an artificial intelligence (AI) system responsible for selecting methods and performing tasks, such as data transformation, data pre-processing, feature selection, model selection and results visualization. Furthermore, the system is in charge of selecting which of their hyper-parameters to optimize. The combination of methods used and their corresponding hyper-parameters is defined as a configuration and these methods are applied using the 10-fold cross validation protocol. Thus, JADBio produces thousands of different models, ranking them based on a scoring metric, in our case, area under the receiver operating characteristic (ROC) curve (AUC), and outputs the best performing one. To eliminate the possibility of overestimating the final predictive performance, JADBio uses a bootstrap-based method to correct it [24]. Using the same method, it calculates the confidence intervals of the resulted performance.

In our analysis, we used JADBio for binary classification modelling for the prediction of the probability of a single individual to achieve DS (PR or SD vs PD) with ICIs as second line treatment. The feature classification of the parameters used as input in JADBio is demonstrated in S1 Table. The tool applied the following modelling algorithms: support vector machines (SVM) with full polynomial and Gaussian kernels [25], random forests [26], ridge logistic regression [27], and decision trees [28]. The performance metric we chose over the several ones available at JADBio, is the AUC. In most cases, the result of an analysis will be a complex model, incomprehensible to the human user. To aid in that regard, JADBio additionally outputs the best interpretable model. In our work, we report the performance estimation of the best performing model.

## Results

### Patient characteristics

Patients’ characteristics are shown in detail in Table 1. All the individuals included in this study were Caucausian. All patients had received prior platinum-based chemotherapy. Median age was 69 years (range: 39–81 years), 24 patients (36.3%) had received steroids, 34 (51.5%) had received ATBs and 22 (33.3% of the total population) had been administered a prolonged course of ATBs. None of the studied clinical and laboratory parameters were associated at a statistical significant level with prolonged ATB administration (S2 Table).

**Table 1:** Baseline patients’ characteristics.

	All patients	
Variable	N	%
**Number of patients**	66	
**Age (years)**		
Median (range)	69 (39–81)	
**Gender**		
Male	55	83.3
Female	11	16.7
**Performance status**		
0–1	51	77.3
2	15	22.7
**Smoking status**		
Active smokers	39	59.1
Former smokers	21	31.8
Never smokers	6	9.1
**Body mass index (BMI)**		
≥ 25 kg/m^2^	32	48.5
< 25 kg/m^2^	34	51.5
**Histology**		
Non-squamous	37	56.1
Squamous	29	43.9
**Number of organs with metastases**		
1–2	45	68.2
>2	21	31.8
**Brain metastases**		
Yes	14	21.2
No	52	78.8
**Liver metastases**		
Yes	19	28.8
No	47	71.2
**Bone metastases**		
Yes	20	30.3
No	46	69.7
**Lymph node metastases**		
Yes	39	59.1
No	27	40.9
**Baseline albumin levels**		
< 3.5 g/dl	12	18.2
≥ 3.5 g/dl	51	77.2
Not available	3	4.5
**Baseline LDH levels**		
> UNL	20	30.3
≤ UNL	36	54.5
Not available	10	15.2
**PDL1 levels**		
< 1%	12	18.2
≥ 1%	20	30.3
Not available	34	51.5
**Steroid administration <10mg of daily prednisolone equivalent for more than 10 days within 15 days before initiation of immunotherapy or during the course of it (first 12 weeks)**		
Steroids naïve	42	63.6
Steroids due to irAEs	8	12.1
Steroids for supportive reasons	16	24.2
**Antibiotics administration within 30 days before the initiation of immunotherapy or during the course of it (first 12 weeks)**		
Yes	34	51.5
No	32	48.5
**Duration of antibiotics administration (days)**		
Median (range)	5 (0–37)	
**Prolonged administration of antibiotics ≥ 14 days within 30 days before the initiation of immunotherapy or during the course of it (first 12 weeks)**		
Yes	22	33.3
No	44	66.7
**Use of inhalation steroids for ≥ 3months before the initiation of immunotherapy**		
Yes	10	15.2
No	56	84.8
**Use of proton pump inhibitors for ≥ 3 months before the initiation of immunotherapy**		
Yes	23	34.8
No	43	65.2
**Grade III or IV iRAEs**		
Yes	8	12.1
No	58	87.9
**Response to immunotherapy**		
CR	0	0
PR	10	15.2
SD	23	34.8
PD	33	50.0
**Disease progression**		
Yes	55	83.3
No	11	16.7
**Death**		
Yes	48	72.7
No	18	27.3
**Duration of response (months)**		
Median (range)	7.97 (2.8–26.9)	
**Progression free survival (months)**		
Median (range)	3.50 (0.16–26.9)	
**Overall survival (months)**		
Median (range)	6.77 (0.6–26.9)	
**Follow up (months)**		
Median (range)	6.37 (0.6–26.9)	

### Effect of the studied variables on response outcomes

Ten (15.2%) patients experienced PR, 23 (34.8%) SD and 33 (50%) had PD at the time of their first disease evaluation. Median duration of response was 7.97 months (range, 2.8–26.9 months).

**Fig 1:**
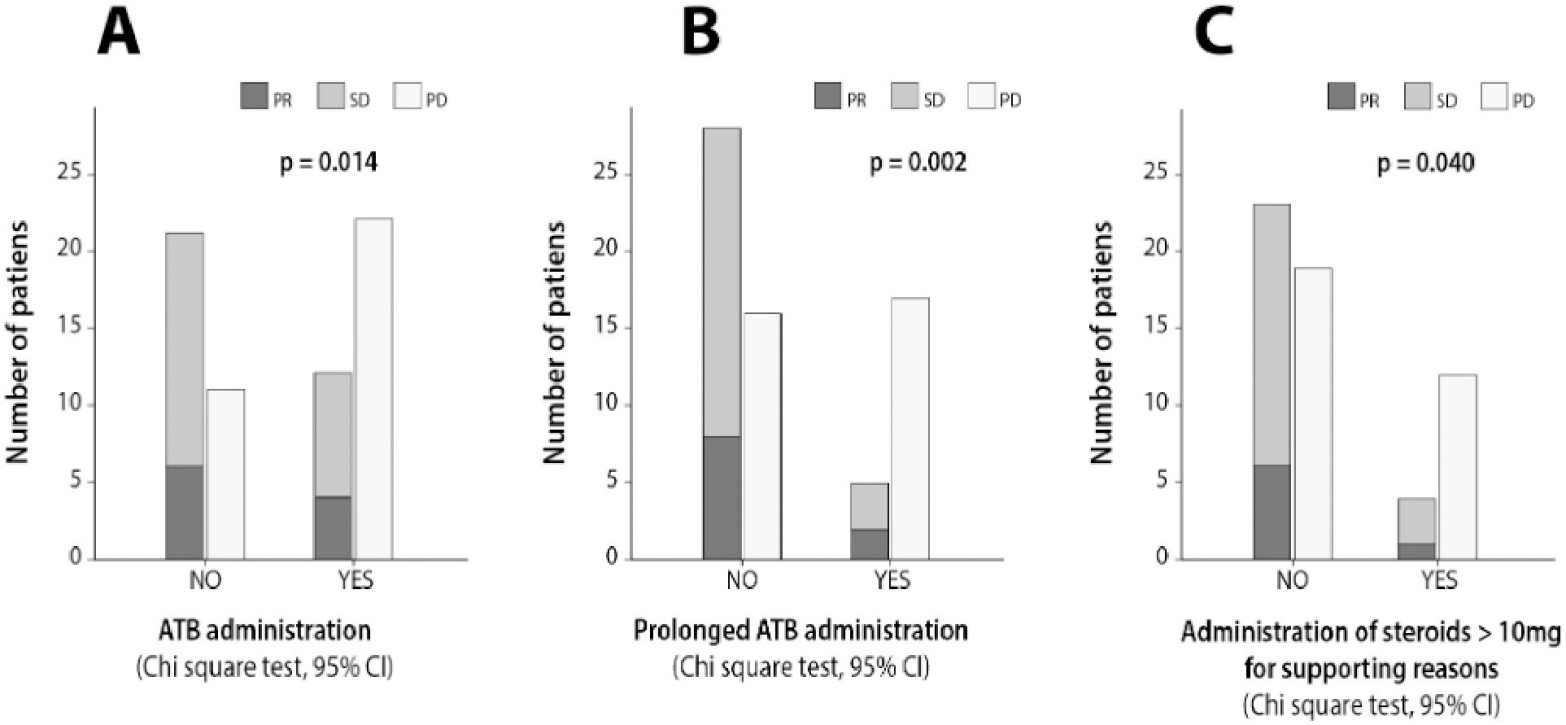
Bar plots depicting the effect of (A) ATB administration (B) prolonged ATB administration and (C) steroid administration >10 mg on disease stabilization rates (PR or SD; Chi-square test, 95%).

Only low BMI (p = 0.030, CI 95%) was significantly associated with inferior response rates (S3 Table). Low BMI (p = 0.003, CI 95%), the presence of bone metastases (p = 0.007, CI 95%), liver metastases (p = 0.014, CI 95%) and high disease burden (p = 0.017, CI 95%) were significantly associated with inferior DS rates. ATB administration (p = 0.014, CI 95%), prolonged ATB administration (p = 0.002, CI 95%) and the use of pos or iv steroids for supportive reasons (p = 0.040, CI 95%), exhibited a statistically significant correlation with reduced DS rates (Fig 1A–1C). The duration of ATB administration in days as a continuous nominal variable was also negatively correlated with DS rates (p = 0.004, CI 95%) (S1 Fig). None of the other studied parameters affected DS rates at a significant level (S4 Table).

**Fig 2:**
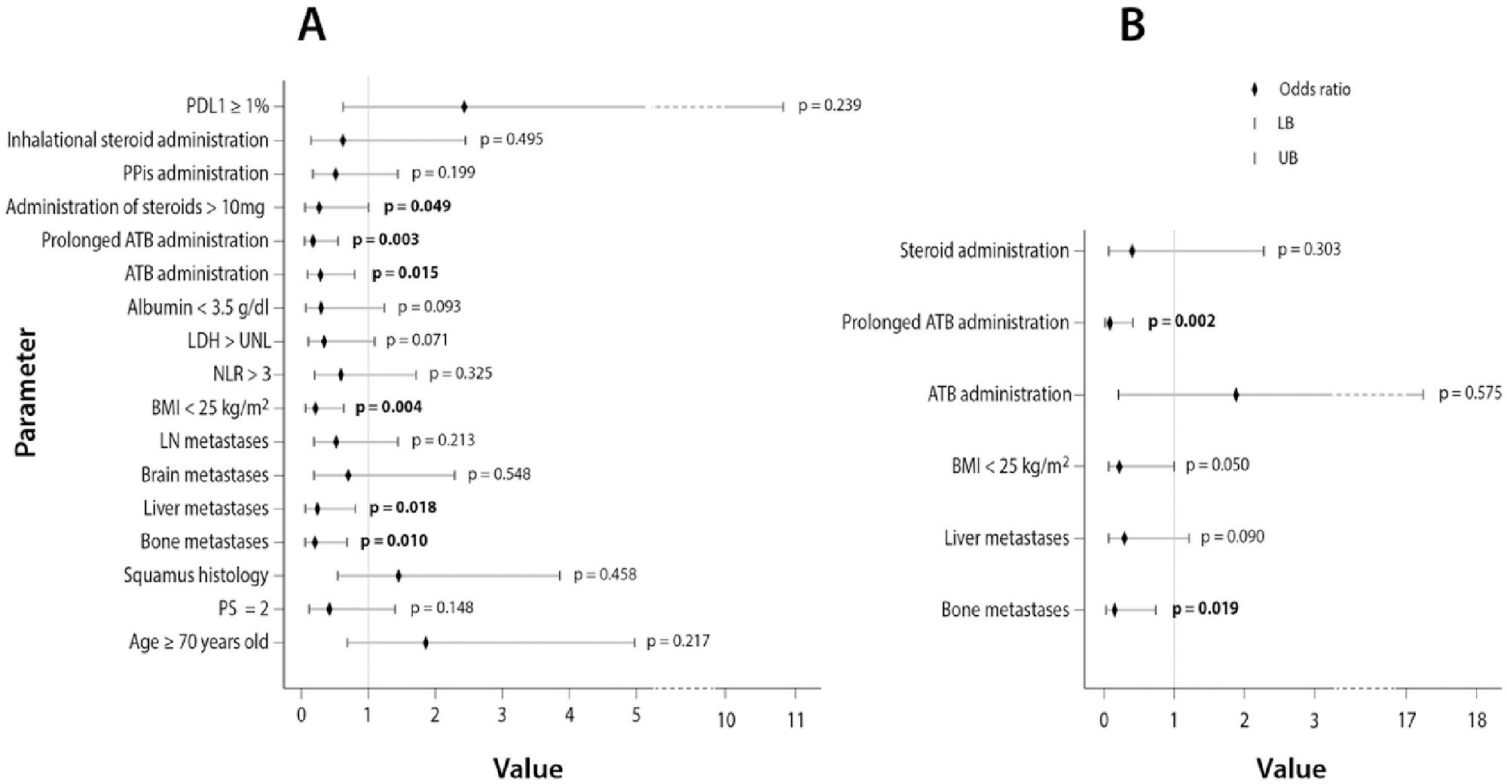
Forest plot depicting the odds ratios of the studied parameters for disease stabilization (PR or SD) in (A) univariate binary regression analysis and (B) multivariate binary regression analysis that included the variables that reached statistical significance (p<0.05) in the univariate analysis. (LB: Lower border, UB: Upper border).

The odds ratio (OR) of each studied covariate on the probability of achieving DS as result of ICIs administration along with its statistical significance are depicted in Fig 2A and S5 Table. In the multivariate logistic regression analysis, bone metastases [OR: 0.153 (CI: 0.032–0.734, p = 0.019)] and prolonged ATB administration [OR: 0.085 (CI: 0.017–0.411, p = 0.002)], independently predicted for lower probability of DS with ICIs (Fig 2B and S5 Table).

### Effect of the studied variables on survival outcomes

Median duration of follow up was 6.37 months (range: 0.6–26.9 months). After data censoring, median PFS and OS for the whole patient population were 3.50 (95% CI: 1.49–5.50) and 6.77 (95% CI: 2.29–11.24) months, respectively.

**Fig 3:**
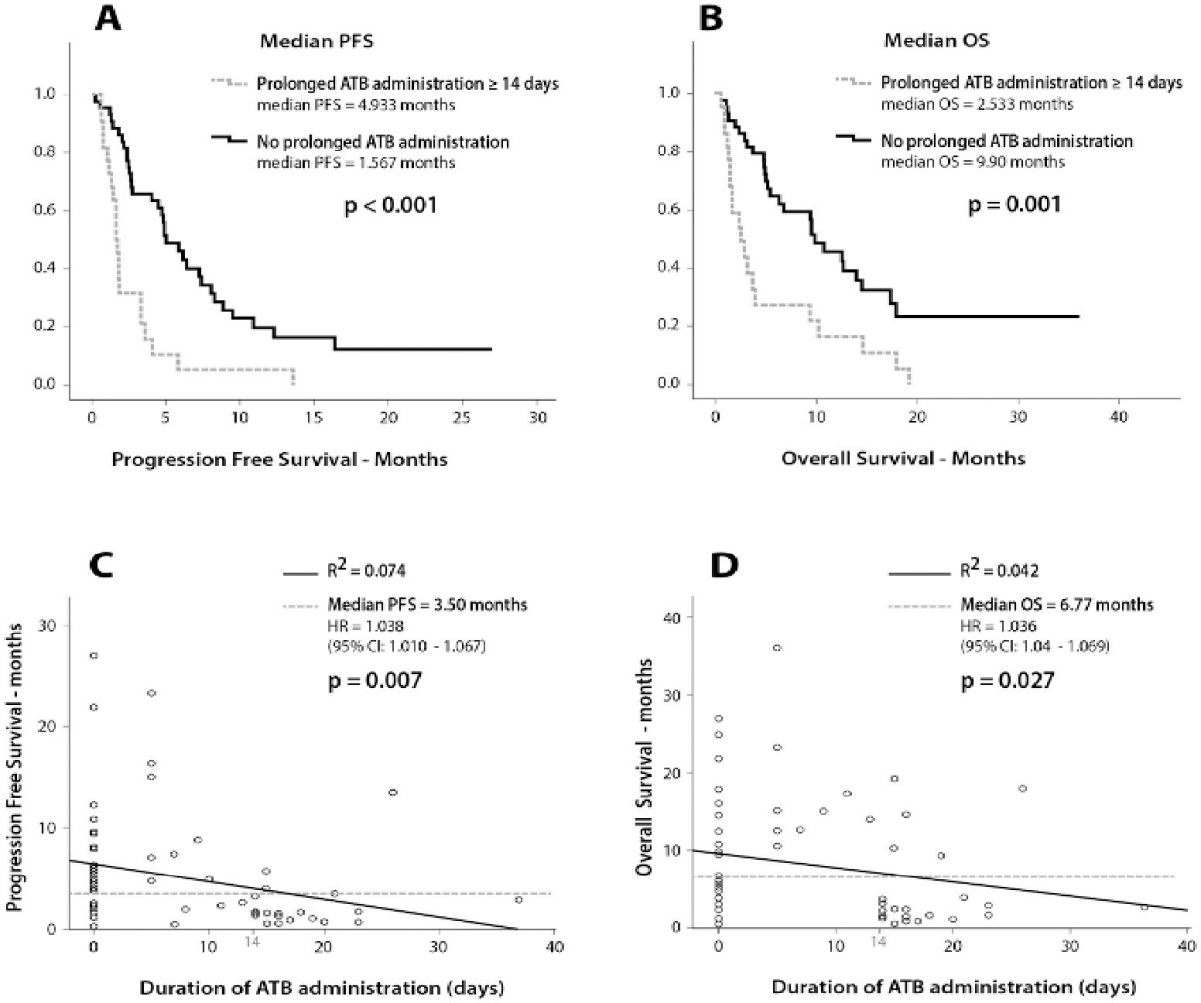
Survival analysis using Kaplan Meier and Cox regression. (A) Effect of prolonged ATB administration on PFS (B) Effect prolonged ATB administration on OS (C) Scatter plot depicting the effect of the duration of ATB administration in days as a continuous variable on PFS (D) Scatter plot depicting the effect of the duration of ATB administration in days as a continuous variable.

The results on the effect of the studied variables on PFS and OS are depicted in S6 Table. BMI <25 kg/m2 (2.33 vs 4.93 months, p = 0.009), high disease burden (1.77 vs 4.67 months, p = 0.008) and the presence of liver (1.73 vs 4.80 months, p = 0.002) or bone metastases (2.10 vs 4.80 months, p = 0.024) were significantly associated with reduced PFS. In addition, baseline albumin levels < 3.5 g/dl (1.70 vs 4.40 months, p = 0.005) and baseline NLR >3 (2.53 vs 4.93, p = 0.024) were also correlated with reduced PFS. Although ATB administration was not associated with lower PFS, (p = 0.062) (S2A Fig), prolonged ATB course (Fig 3A) and steroid administration > 10 mg for supportive reasons (S3A Fig) were significantly correlated with inferior PFS (1.57 vs 4.93 months, p<0.001 and 1.27 vs 4.70 months, p = 0.013). None of the other analyzed covariates exhibited statistically significant correlations with PFS (S4A and S5A Figs and S6 Table).

Regarding their effect on patients’ survival, PS 2 (3.17 vs 9.60 months, p = 0.027), baseline albumin levels < 3.5 g/dl (1.70 vs 9.57 months, p = 0.003), baseline LDH levels > UNL (3.70 vs 9.90 months, p = 0.040) and the presence of bone metastases (3.77 vs 10.33 months, p = 0.011) exhibited a negative correlation with OS (S6A and S6B Fig). Prolonged ATB administration was associated with reduced OS (2.50 vs 9.93 months, p = 0.001) (Fig 3B), however, the use of steroids (2.53 vs 9.60 months, p = 0.051) or of ATB (4.00 vs 9.67 months, p = 0.301) (S2B and S3B Figs) were not correlated with reduced OS. No other associations with inferior OS were observed (S4B and S5B Figs and S6 Table).

### Univariate and multivariate survival analysis

Cox Regression analysis revealed that the duration of ATB administration evaluated as a continuous nominal variable was negatively correlated with PFS (p = 0.007, CI 95%) and OS (p = 0.027, 95% CI) (Fig 3C and 3D).

In the univariate analysis for PFS, baseline albumin levels were not included in the analysis due to insufficient number of events; results are shown in Table 2. Multivariate analysis demonstrated that steroids used for supportive reasons [HR = 2.556 (CI: 1.347–4.887, p = 0.004)], prolonged administration of ATBs [HR = 3.403 (CI: 1.817–6.375, p = 0.0001)] and the presence of liver [HR = 3.266 (CI: 1.653–6.375, p = 0.001)] or bone metastases [HR = 2.244 (CI: 1.155–4.360, p = 0.017)] were independent predictors for inferior PFS (Table 2).

**Table 2:** Univariate and multivariate analysis using Cox Regression Method.

Cox regression	PFS		OS	
**Univariate analysis**	**HR (95% CI)**	***p* value**	**HR (95%CI)**	***p* value**
Performance status	1.574(0.855–2.899)	0.145	1.999(1.068–3.740)	**0.030**
Age ≥ 70 years old	1.127(0.661–1.922)	0.660	1.193(0.673–2.114)	0.546
Smoker of former smoker	1.126(0.404–3.135)	0.821	2.361(0.571–9.757)	0.235
Female gender	1.033(0.504–2.120)	0.929	1.144(0.535–2.445)	0.729
Brain metastases	1.242(0.653–2.364)	0.509	1.022(0.493–2.118)	0.953
Bone metastases	1.913(1.078–3.394)	**0.027**	2.135(1.171–3.893)	**0.013**
Liver metastases	2.503(1.390–4.506)	**0.002**	1.443(0.781–2.665)	0.241
Disease burden	2.115(1.201–3.725)	**0.009**	1.562(0.860–2.840)	0.142
Steroid administration > 10 mg	2.156(1.158–4.013)	**0.015**	1.908(0.985–3.698)	0.055
ATB^a^ administration	1.655(0.068–2.830)	0.065	1.353(0.761–2.406)	0.304
Prolonged ATB administration ≥ 14 days	3.181(1.795–5.637)	**0.0001**	2.646(1.476–4.741)	**0.001**
NLR^b^	1.939(1.050–3.559)	**0.033**	1.588(0.855–2.947)	0.143
LDH>UNL	1.674(0.935–2.997)	0.083	1.868(1.018–3.425)	**0.044**
**Multivariate analysis**	**HR (95% CI)**	***p* value**	**HR (95%CI)**	***p* value**
Performance status			1.878(0.963–3.661)	0.075
Bone metastasis	2.244(1.155–4.360)	**0.017**	1.890(1.017–3.512)	**0.049**
Liver metastasis	3.266(1.653–6.375)	**0.001**		
Disease burden	1.552(0.555–4.329)	0.401		
Steroid administration > 10 mg	2.566(1.347–4.887)	**0.004**		
Prolonged ATB administration ≥ 14 days	3.403(1.817–6.375)	**0.0001**	2.945(1.619–5.358)	**0.0001**
NLR	1.147(0.580–2.269)	0.693		
LDH > UNL^c^			1.618(0.877–2.985)	0.123

^a^: ATB = Antibiotics,

^b^: NRL = Neutrophil to Lymphocyte ratio,

^c^: UNL = Upper normal limit (247 Units/liter).

In the multivariate analysis for OS, prolonged use of ATBs [HR = 2.945 (CI: 1.619–5.358, p = 0.0001)] and bone metastases [HR = 1.890 (CI: 1.017–3.512, p = 0.049)] were independently associated with reduced survival (Table 2).

### Multivariate analysis using the JADBio tool

**Fig 4:**
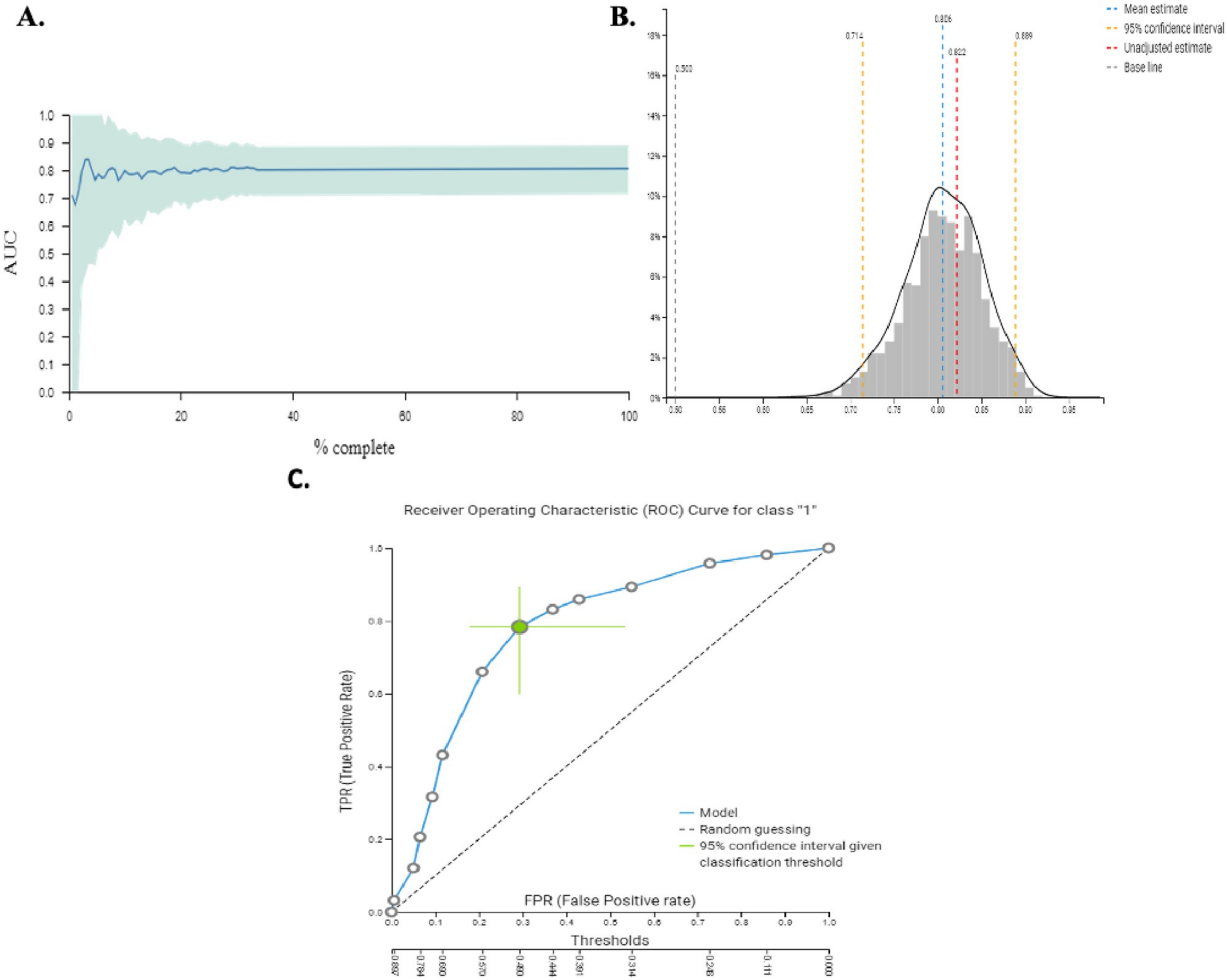
A. Visualization of the learning process of JADbio for the classification analysis of our data. JADbio tried 3017 configurations and trained 211190 models in total. B. Distribution of the performance metric (AUC) of our model. The distribution is computed on out of sample predictions of the current model. C. Receiver Operating Characteristic (ROC) Curve for the best performing model (Support Vector Machines (SVM) of type C-SVC with Polynomial Kernel and hyper-parameters: cost = 0.01, gamma = 1.0, degree = 4). The classification threshold for the 95% confidence intervals has been set at the average F1/accuracy/Balance accuracy.

**Fig 5:**
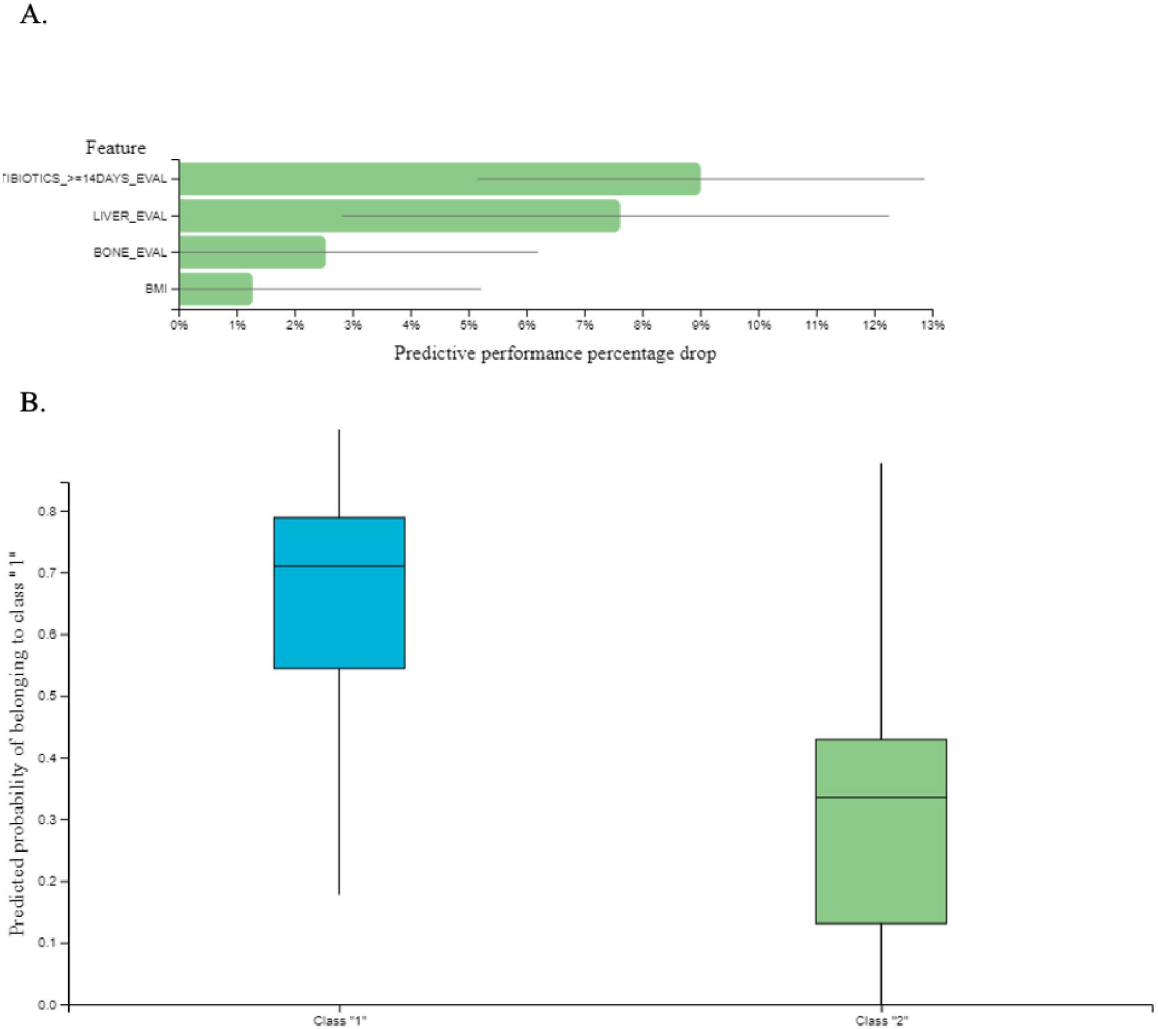
A. Feature importance plot: This chart reports feature importance defined as the percentage drop in predictive performance when the feature is removed from the model. Grey lines indicate 95% confidence intervals. B. The Box-Plot contrasts the cross-validated predicted probability of belonging to a specific class against the actual class of the samples. Well-performing models are expected to provide predictions that are close to 1 for the actual class and close to 0 for all other class. Class 1 is the probability of achieving PR or SD as response to immunotherapy and class 2 is the probability of developing disease progression.

For the classification task (response to ICIs), patients were divided into two groups; responders were characterized as those experiencing PR or SD and non-responders were those experiencing PD as best response to treatment. For the classification analysis, JADbio tried 3017 configurations and trained 211190 models (Fig 4A). JADbio performed LASSO feature selection (penalty = 0.5, lambda = 0.027) and selected 4 features: prolonged ATB administration, bone metastases, liver metastases and BMI < 25 kg/m^2^ for the original signature. In total there was only one signature. The predictive algorithm of the best performing model was SVM of type C-SVC with Polynomial Kernel and hyper-parameters: cost = 0.01, gamma = 1.0, degree = 4 with an AUC = 0.806 [0.714–0.889] (Fig 4B). The ROC curve of the best performing model is demonstrated in Fig 4C. In addition, the classification analysis was able to calculate the feature importance of the selected features on the probability of achieving PR or SD which was defined as the percentage drop in predictive performance when each particular feature was removed from the model (Fig 5A). The box plots that visualize the contrast of the cross-validated predicted probability of belonging to a specific class against the actual class of the samples are depicted in Fig 5B.

## Discussion

The primary aim of our study was to prospectively evaluate the effect of common clinical and laboratory parameters on the outcome of patients with metastatic NSCLC receiving immunotherapy. We herein demonstrate that routinely available patient and disease characteristics are correlated with treatment outcomes and can be integrated into a multifactorial model predicting individual clinical benefit from ICIs using a machine learning approach.

In our cohort, bone metastases constituted an adverse prognostic factor in NSCLC patients treated with second line ICIs. In accordance, previous retrospective studies highlight that ICI efficacy may vary according to the presence of specific disease sites [29, 30]. Furthermore, we observed that patients with low BMI had shorter PFS compared to patients with high ΒΜΙ. Low albumin levels were also significantly associated with both shorter PFS and OS in our cohort. However, it should be noted that BMI and/or albumin levels cannot be used alone for the evaluation of patients’ nutritional status. More detailed analysis is required to further elucidate the impact of body composition in the outcome of cancer patients treated with immunotherapy [31, 32]. Performance status 2 did not emerge as an independent negative prognostic factor, however this could be attributed to the small sample size of our cohort and the fact that only 15 patients had performance status 2, leading to increased probability for a statistical type I error.

The administration of commonly used co-medications exerted a significant impact on patients’ outcome in our analysis. The time frame of 12 weeks from the start of ICIs initiation employed for the classification of patients according to the use of steroids and ATBs was set to avoid a bias for those achieving long term clinical remissions, who thus, would have a higher possibility for steroid and ATB use during the course of their illness. ATB administration as a categorical variable did not affect PFS or OS, however when we examined the effect of their administration in days as a continuous nominal variable, a statistically significant negative correlation with PFS and OS was revealed. Importantly, prolonged ATBs administration emerged as an independent predictor for reduced probability of disease stabilization and for inferior PFS and OS. Our results are in accordance with previously published prospective and retrospective studies [33, 34] and further reinforce the data published by Tinsley et al [35] reporting worst outcomes with cumulative ATB administration in patients with metastatic malignancies receiving ICIs indicating a dose dependent effect between ATB exposure and reduced ICI efficacy. Our findings along with the results from previously published reports suggest that ATB administration, especially for longer time periods should be avoided in patients receiving immunotherapy.

In our analysis we classified patients who received steroids into two different subgroups, those who were administered steroids for supportive reasons and those who received high dose steroids due to immune related adverse effects (irAEs). In our cohort, the use of steroids for supportive reasons was independently associated with shorter PFS, however no associations with OS were revealed. Regrettably, due to the small number of patients we were not able to perform more detailed subgroup analysis regarding the specific reasons for the supportive administration of steroids, however, our findings reinforce previous reports raising concerns regarding the use of > 10 mg prednisone equivalent in patients receiving ICIs [36]. On the contrary, the administration of inhalational steroids or of PPis did not impair treatment outcomes in our analysis and their administration seems safe. Our results regarding the effects of PPis are in contrast with a recent published retrospective analysis by Hopkins et al [37] demonstrating that PPis negatively affected survival outcomes in individuals with metastatic urothelial cancer treated with atezolizumab. Therefore, further research is needed in order to clarify their effects on the efficacy of ICIs.

We further incorporated all the recorded parameters into JADbio, an artificial intelligence (AI) system, with to evaluate how these different features interact with one another and to examine the feature importance of each particular parameter on individual treatment outcomes in an integrative manner. JADbio selected four clinical features and created a signature that was able to predict the possibility for disease stabilization as result of treatment with ICIs with an accuracy of 81%. These four features consisted of prolonged ATB administration, bone metastases, liver metastases and BMI < 25 kg/m^2^. The higher feature importance obtained for prolonged ATB administration further underscores the importance of the microbiome composition for an effective antitumor immune response [16]. Interestingly, they indicate that factors likely inherent to the biology of the primary tumour are related to both the specific patterns of metastatic spread and the response to ICIs.

Previous studies have employed AI for the prediction of outcome of patients with metastatic melanoma and NSCLC receiving treatment with ICIs [38, 39]. Despite the significant smaller sample of the cohort studied herein, our analysis provides the advantage of including a wider range of routinely available, easily obtainable clinical parameters and more importantly it allows the calculation of each parameter’s feature importance on the determination of an individual’s outcome. Using JADbio, we constructed a novel multivariate model predicting the individual possibility of achieving disease stabilization in patients with metastatic NSCLC treated with ICIs in the second line setting with accuracy at the level of 81%. Using other AutoML services, an overestimation of the final predictive performance might have resulted, as they mainly use cross-validation, which overfits in sample-sample scenarios [40]. JADBio is built for such scenarios and uses a technique [24] to remove this cross-validation bias, rendering the final performance estimation conservative and reliable.

Our results indicate that routinely available variables could be used to identify individuals who will achieve disease control with ICIs and to spot those who will likely progress on treatment for whom closer monitoring, alternative treatment regimens or participation in clinical trials should be advised. However, it should be also noted that immunotherapy has now moved into the first line treatment setting either as a monotherapy or in combination with chemotherapy and that only a minority of patients now receive ICIs in the second line or beyond.

Strengths of our study include the prospective evaluation and analysis of a wide range of common and easily accessible clinical and laboratory parameters and of common co-medications in a cohort of lung cancer patients treated with immunotherapy to determine their prognostic role. A significant novelty of our report represents the additional multivariate analysis using an AutoML interface to further determine the integrated feature importance of each of these parameters on individual patient outcomes. Limitations of our study are the small statistical sample and the fact that PD-L1 levels were not included in our model due to high rates of missing data. In addition, our results were not validated in another patient cohort.

## Conclusion

Our results corroborate previous evidence regarding the negative predictive role of liver and bone metastases in NSCLC patients treated with ICIs. Furthermore, they emphasize the negative effect of ATBs on patient outcomes and suggest that long-term ATBs use should be avoided in these patients. By incorporating our data into the JADbio machine learning system, we were able to distinguish the clinical variables that are most relevant for achieving disease control with immunotherapy. Importantly, we estimated the feature importance of these variables on individual patient outcomes in an integrative manner. Since immunotherapy is currently mostly used either as first line treatement, either as a single agent or in combination with chemotherapy, our findings merit further evaluation in these settings.

## Supporting information

S1 Table Binary classification of patients’ feature input in JADBio. (DOC)

S2 Table Chi square test demonstrating the association of the analyzed clinical parameters with a prolonged ATB course. (DOC)

S3 Table Effect of the studied variables on response rates. (DOC)

S4 Table Effect of the studied variables on disease stabilization rates. (DOC)

S5 Table Univariate and multivariate logistic regression on the Odds Ratio (OR) of the analyzed covariates on the probability of achieving disease stabilization (PR or SD) as response to treatment with ICIs. (DOC)

S6 Table Kaplan Meier method (log-rank test) on the effect of the studied parameters on PFS and OS. (DOC)

S1 Fig Mann-Whitney U test examining the effect of the duration of ATB administration in days on DS rates. On the left side are the days on ATB of the patients that experienced PD and on the right side the days on ATB of those who had DS (PR or SD) as response to ICI administration.
(DOC)

S2 Fig Kaplan-Meier curves on the effect of ATB administration on PFS (A) and OS (B). (DOC)

S3 Fig Kaplan-Meier curves on the effect of steroid administration > 10 mg on PFS (A) and OS (B). (DOC)

S4 Fig Kaplan-Meier curves on the effect of chronic PPis administration on (A) PFS and (B) OS. (DOC)

S5 Fig Kaplan-Meier curves on the effect of inhalational steroids administration on (A) PFS and (B). (DOC)

S6 Fig Kaplan Meier curves depicting the effect of the following parameters on OS (A) Baseline albumin levels <3.5 g/dl (B) Presence of bone metastases. (DOC)
