# Correction: Prediction of outcome in patients with non-small cell lung cancer treated with second line PD-1/PDL-1 inhibitors based on clinical parameters: Results from a prospective, single institution study

**DOI:** 10.1371/journal.pone.0294382

**Published:** 2023-11-09

**Authors:** Konstantinos Rounis, Dimitrios Makrakis, Chara Papadaki, Alexia Monastirioti, Lambros Vamvakas, Konstantinos Kalbakis, Krystallia Gourlia, Iordanis Xanthopoulos, Ioannis Tsamardinos, Dimitrios Mavroudis, Sofia Agelaki

An additional affiliation is missing for the first author. Konstantinos Rounis is also affiliated with: Laboratory of Translational Oncology, Medical School, University of Crete.
