# Supplementary material for: Prediction of outcome in patients with non-small cell lung cancer treated with second line PD-1/PDL-1 inhibitors based on clinical parameters: Results from a prospective, single institution study
Source: PLoS One. 2021 Jun 1;16(6):e0252537. doi: 10.1371/journal.pone.0252537 (PMC8168865; doi:10.1371/journal.pone.0252537)
Supplement: S1 Table — (DOC) [file pone.0252537.s001.doc]

**S1 Table: Binary classification of patients’ feature input in JADBio.**

| **Parameter** | **Feature** | **JADBio input** |
| --- | --- | --- |
| Female gender | Yes | 1 |
|  | No | 2 |
| Performance status 0-1 | Yes | 1 |
|  | No | 2 |
| Age ≥ 70 years old | Yes | 1 |
|  | No | 2 |
| BMI < 25 kg/m^2^ | Yes | 1 |
|  | No | 2 |
| Non-squamous histology | Yes | 1 |
|  | No | 2 |
| Presence of brain metastases | Yes | 1 |
|  | No | 2 |
| Presence of liver metastases | Yes | 1 |
|  | No | 2 |
| Presence of lung or pleural metastases | Yes | 1 |
|  | No | 2 |
| Presence of bone metastases | Yes | 1 |
|  | No | 2 |
| Presence of LN metastases | Yes | 1 |
|  | No | 2 |
| LDH ≤ UNL | Yes | 1 |
|  | No | 2 |
| Albumin < 3.5 g/dl | Yes | 1 |
|  | No | 2 |
| Baseline NLR > 3 | Yes | 1 |
|  | No | 2 |
| ATB administration | Yes | 1 |
|  | No | 2 |
| Prolonged ATB administration ≥ 14 days duration | Yes | 1 |
|  | No | 2 |
| Steroid administration > 10 mg for ≥ 10 days | Yes | 1 |
|  | No | 2 |
| Chronic PPis administration | Yes | 1 |
|  | No | 2 |
| Chronic administration of inhalational steroids | Yes | 1 |
|  | No | 2 |
| PR or SD to 1^st^ line platinum doublet | Yes | 1 |
|  | No | 2 |
| PR or SD to immunotherapy | Yes | 1 |
|  | No | 2 |

Abbreviations: BMI=Body mass index, LDH=Lactate dehydrogenase, UNL=Upper normal limit (247 units/liter), NLR=Neutrophile to lymphocyte ratio, PR=Partial response, SD=Disease stabilization, ATB=Antibiotics, PPis=Proton pump inhibitors
