# Supplementary material for: Prediction of outcome in patients with non-small cell lung cancer treated with second line PD-1/PDL-1 inhibitors based on clinical parameters: Results from a prospective, single institution study
Source: PLoS One. 2021 Jun 1;16(6):e0252537. doi: 10.1371/journal.pone.0252537 (PMC8168865; doi:10.1371/journal.pone.0252537)
Supplement: S2 Table — (DOC) [file pone.0252537.s002.doc]

**S2 Table: Chi square test demonstrating the association of the analyzed clinical parameters with a prolonged ATB course.**

| **Variable** | **N=66** | **Prolonged ATB^a^-** | **Prolonged ATB+** | | ***P* value**  **(chi-square test, 95% CI)** |
| --- | --- | --- | --- | --- | --- |
| **BMI^b^ < 25 kg/m^2^** |  | | | | |
| Yes | 34 | 19 | 15 | | p=0.055 |
| No | 32 | 25 | 7 | |
| **Liver metastases** |  | | | | |
| Yes | 19 | 12 | 7 | | p=0.701 |
| No | 47 | 32 | 15 | |
| **Brain metastases** |  | | | | |
| Yes | 14 | 7 | 7 | | p=0.136 |
| No | 52 | 37 | 15 | |
| **Bone metastases** |  | | | | |
| Yes | 20 | 15 | 5 | | p=0.344 |
| No | 46 | 29 | 17 | |
| **Performance status** |  | | | | |
| 0-1 | 51 | 36 | 15 | | p=0.213 |
| 2 | 15 | 8 | 7 | |
| **LDH^c^ levels>UNL^d^** | N=56 | | | | |
| Yes | 20 | 11 | 9 | | p=0.280 |
| No | 36 | 25 | 11 | |
| **Albumin < 3.5 g/dl** | N=63 | | | | |
| Yes | 12 | 7 | 5 | p=0.496 | |
| No | 51 | 35 | 16 |
| **NLR^e^>3** | N=62 | | | | |
| Yes | 41 | 27 | 14 | | p=0.657 |
| No | 21 | 15 | 6 | |
| **PDL1^f^ ≥ 1%** | N=32 | | | | |
| Yes | 20 | 15 | 5 | | p=0.325 |
| No | 12 | 7 | 5 | |

a: ATB=Antibiotics, b: BMI=Body mass index, c: LDH=Lactate dehydrogenase, d: UNL=Upper normal limit (247 units/liter), e: NLR=Neutrophil to lymphocyte ratio, f: PDL1=Programmed death ligand 1
