# Supplementary material for: Prediction of outcome in patients with non-small cell lung cancer treated with second line PD-1/PDL-1 inhibitors based on clinical parameters: Results from a prospective, single institution study
Source: PLoS One. 2021 Jun 1;16(6):e0252537. doi: 10.1371/journal.pone.0252537 (PMC8168865; doi:10.1371/journal.pone.0252537)
Supplement: S3 Table — (DOC) [file pone.0252537.s003.doc]

**S3 Table: Effect of the studied variables on response rates.**

| **Variable** | **N=66** | **PR** | **SD or PD** | | ***P* value**  **(chi-square test, 95% CI)** |
| --- | --- | --- | --- | --- | --- |
| **ATB^a^ administration** |  |  | | | |
| Yes | 34 | 4 | 30 | | P=0.429 |
| No | 32 | 6 | 26 | |
| **Prolonged ATB administration** |  | | | | |
| Yes | 22 | 2 | 20 | | P=0.332 |
| No | 44 | 8 | 36 | |
| **Baseline Steroid administration > 10 mg ≥ 10 day** | N=58 |  | | | |
| Yes | 16 | 1 | 15 | | P=0.401 |
| No | 42 | 6 | 36 | |
| **Use of inhalational steroids** |  | | | | |
| Yes | 10 | 2 | 8 | | P=0.642 |
| No | 56 | 8 | 48 | |
| **PPis^b^ administration** |  | | | | |
| Yes | 23 | 3 | 20 | | P=0.727 |
| No | 43 | 7 | 36 | |
| **BMI^c^ < 25 kg/m^2^** |  | | | | |
| Yes | 34 | 2 | 32 | | P=**0.030** |
| No | 32 | 8 | 24 | |
| **Liver metastases** |  | | | | |
| Yes | 19 | 1 | 18 | | P=0.154 |
| No | 47 | 9 | 38 | |
| **Brain metastases** |  | | | | |
| Yes | 14 | 2 | 12 | | P=0.919 |
| No | 52 | 8 | 44 | |
| **Bone metastases** |  | | | | |
| Yes | 20 | 2 | 18 | | P=0.442 |
| No | 46 | 8 | 38 | |
| **LN^d^ metastases** |  | | | | |
| Yes | 39 | 7 | 32 | | P=0.446 |
| No | 27 | 3 | 24 | |
| **Disease burden^e^** |  | | | | |
| High | 21 | 2 | 19 | | P=0.384 |
| Low | 45 | 8 | 37 | |
| **Performance status** |  | | | | |
| 0-1 | 51 | 43 | 8 | | P=0.823 |
| 2 | 15 | 2 | 13 | |
| **LDH^f^ levels>UNL^g^** | N=56 | | | | |
| Yes | 20 | 2 | 18 | | P=0.898 |
| No | 36 | 4 | 32 | |
| **Albumin < 3.5 g/dl** | N=63 | | | | |
| Yes | 12 | 0 | 12 | p=0.173 | |
| No | 51 | 7 | 47 |
| **NLR^h^>3** | N=62 | | | | |
| Yes | 41 | 3 | 38 | | P=0.167 |
| No | 21 | 4 | 17 | |
| **PDL1^i^ ≥ 1%** | N=32 | | | | |
| Yes | 20 | 3 | 17 | | P=0.581 |
| No | 12 | 1 | 11 | |

a: ATB=Antibiotics, b: PPis=Proton pump inhibitors, c: BMI=Body mass index, d: LN=Lymph nodes, e: Disease burden high=More than 2 organs affected with metastatic disease, f: LDH=Lactate dehydrogenase, g: UNL=Upper normal limit (247 units/liter), h: NLR=Neutrophil to lymphocyte ratio, i: PDL1=Programmed death ligand 1
