# Supplementary material for: Prediction of outcome in patients with non-small cell lung cancer treated with second line PD-1/PDL-1 inhibitors based on clinical parameters: Results from a prospective, single institution study
Source: PLoS One. 2021 Jun 1;16(6):e0252537. doi: 10.1371/journal.pone.0252537 (PMC8168865; doi:10.1371/journal.pone.0252537)
Supplement: S4 Table — (DOC) [file pone.0252537.s004.doc]

**S4 Table: Effect of the studied variables on disease stabilization rates.**

| **Variable** | **N=66** | **PR or SD** | **PD** | | ***P* value**  **(chi-square test,95% CI)** |
| --- | --- | --- | --- | --- | --- |
| **ATB^a^ administration** |  |  | | | |
| Yes | 34 | 12 | 22 | | P=**0.014** |
| No | 32 | 21 | 11 | |
| **Prolonged ATB administration** |  | | | | |
| Yes | 22 | 5 | 17 | | P=**0.002** |
| No | 44 | 28 | 16 | |
| **Baseline Steroid administration > 10 mg ≥ 10 day** | N=58 |  | | | |
| Yes | 16 | 4 | 12 | | P=**0.040** |
| No | 42 | 23 | 19 | |
| **Use of inhalational steroids** |  | | | | |
| Yes | 10 | 4 | 6 | | P=0.367 |
| No | 56 | 29 | 27 | |
| **PPis^b^ administration** |  | | | | |
| Yes | 23 | 9 | 14 | | P=0.151 |
| No | 43 | 14 | 19 | |
| **BMI^c^ < 25 kg/m^2^** |  | | | | |
| Yes | 34 | 11 | 23 | | P=**0.003** |
| No | 32 | 22 | 10 | |
| **Liver metastases** |  | | | | |
| Yes | 19 | 5 | 14 | | P=**0.014** |
| No | 47 | 28 | 19 | |
| **Brain metastases** |  | | | | |
| Yes | 14 | 6 | 8 | | P=0.382 |
| No | 52 | 27 | 25 | |
| **Bone metastases** |  | | | | |
| Yes | 20 | 5 | 15 | | P=**0.007** |
| No | 46 | 28 | 18 | |
| **LN^d^ metastases** |  | | | | |
| Yes | 39 | 22 | 17 | | P=0.158 |
| No | 27 | 11 | 16 | |
| **Disease burden^e^** |  | | | | |
| High | 21 | 6 | 15 | | P=**0.017** |
| Low | 45 | 27 | 18 | |
| **Performance status** |  | | | | |
| 0-1 | 51 | 28 | 23 | | P=0.120 |
| 2 | 15 | 5 | 10 | |
| **LDH^f^ levels>UNL^g^** | N=56 | | | | |
| Yes | 20 | 6 | 14 | | P=0.059 |
| No | 36 | 20 | 16 | |
| **Albumin < 3.5 g/dl** | N=63 | | | | |
| Yes | 12 | 3 | 9 | p=0.076 | |
| No | 51 | 27 | 24 |
| **NLR^h^>3** | N=62 | | | | |
| Yes | 41 | 18 | 23 | | P=0.236 |
| No | 21 | 12 | 9 | |
| **PDL1i ≥ 1%** | N=32 | | | | |
| Yes | 20 | 11 | 9 | | P=0.234 |
| No | 12 | 4 | 8 | |

a: ATB=Antibiotics, b: PPis=Proton pump inhibitors, c: BMI=Body mass index, d: LN=Lymph nodes, e: Disease burden high=More than 2 organs affected with metastatic disease, f: LDH=Lactate dehydrogenase, g: UNL=Upper normal limit (247 units/liter), h: NLR=Neutrophil to lymphocyte ratio, i: PDL1=Programmed death ligand 1
