# Supplementary material for: Prediction of outcome in patients with non-small cell lung cancer treated with second line PD-1/PDL-1 inhibitors based on clinical parameters: Results from a prospective, single institution study
Source: PLoS One. 2021 Jun 1;16(6):e0252537. doi: 10.1371/journal.pone.0252537 (PMC8168865; doi:10.1371/journal.pone.0252537)
Supplement: S5 Table — (DOC) [file pone.0252537.s005.doc]

**S5 Table: Univariate and multivariate logistic regression on the odds ratio (OR) of the analyzed covariates on the probability of achieving disease stabilization (PR or SD) as response to treatment with ICIs.**

|  | **Univariate** | |  | **Multivariate** | |  |
| --- | --- | --- | --- | --- | --- | --- |
| **Variable** | OR (95% CI) | p value | AUC^i^  (95% CI) | OR (95% CI) | p value | AUC  (95% CI) |
| Age ≥ 70 years old | 1.858 (0.694-4.975) | 0.217 | 0.576  (0.437-0.714) |  |  |  |
| PS^a^ = 2 | 0.410 (0.122-1.373) | 0.148 | 0.576  (0.437-0.714) |  |  |  |
| Squamous histology | 1.447 (0.545-3.846) | 0.458 | 0.545  (0.406-0.685) |  |  |  |
| Bone metastases | 0.214 (0.066-0.692) | **0.010** | 0.652  (0.518-0.785) | **0.153** (0.032-0.734) | **0.019** |  |
| Liver metastases | 0.242 (0.074-0.784) | **0.018** | 0.636  (0.501-0.771) | 0.290 (0.069-1.210) | 0.090 |  |
| Brain metastases | 0.694 (0.211-2.283) | 0.548 | 0.530  (0.390-0.670) |  |  |  |
| LN^b^ metastases | 0.531 (0.196-1.436) | 0.213 | 0.576  (0.437-0.714) |  |  |  |
| BMI^c^ < 25 kg/m^2^ | 0.217 (0.077-0.631) | **0.004** | 0.682  (0.551-0.813) | 0.220 (0.069-1.000) | 0.050 |  |
| NLR^d^ > 3 | 0.587 (0.203-1.697) | 0.325 | 0.559  (0.415-0.703) |  |  |  |
| LDH^e^ > UNL^f^ | 0.342 (0.107-1.094) | 0.071 | 0.618  (0.470-0.766) |  |  |  |
| Albumin < 3.5 g/dl | 0.296 (0.071-1.222) | 0.093 | 0.586  (0.445-0.727) |  |  |  |
| ATB^g^ administration | 0.285 (0.103-0.787) | **0.015** | 0.652  (0.518-785) | 1.883 (0.206-17.241) | 0.575 |  |
| Prolonged ATB administration | 0.168 (0.052-0.542) | **0.003** | 0.682  (0.551-0.813) | **0.085** (0.017-0.411) | **0.002** |  |
| Administration of steroids > 10 mg | 0.275 (0.076-0.995) | **0.049** | 0.619  (0.475-0.764) | 0.401 (0.070-2.277) | 0.303 |  |
| PPis^h^ administration | 0.508 (0.181-1.426) | 0.199 | 0.576  (0.437-0.714) |  |  |  |
| Inhalational steroids administration | 0.620 (0.410-2.439) | 0.495 | 0.530  (0.390-0.670) |  |  |  |
| PDL1^i^ ≥ 1% | 2.444 (0.552-10.833) | 0.239 | 0.602  (0.403-0.801) |  |  |  |
| Overall |  |  |  |  |  | 0.845  (0.746-0.944) |

a: PS=Performance status, b: LN=Lymph nodes, c: BMI=Body mass index, d: NLR= neutrophil/lymphocyte ratio, e: LDH=Lactate dehydrogenase, f: UNL=Upper normal limit, g: ATB=Antibiotics, h: PPis=Proton pump inhibitors, i: AUC=Area under the ROC curve
