# Supplementary material for: Prediction of outcome in patients with non-small cell lung cancer treated with second line PD-1/PDL-1 inhibitors based on clinical parameters: Results from a prospective, single institution study
Source: PLoS One. 2021 Jun 1;16(6):e0252537. doi: 10.1371/journal.pone.0252537 (PMC8168865; doi:10.1371/journal.pone.0252537)
Supplement: S6 Table — (DOC) [file pone.0252537.s006.doc]

**S6 Table: Kaplan Meier method (log-rank test) on the effect of the studied parameters on PFS and OS**

|  | **Median PFS**  **(months)** | **p value**  **(log-rank test)** | **Median OS**  **(months)** | **p value**  **(log-rank test)** |
| --- | --- | --- | --- | --- |
| All patients (n=66) | 3.50 |  |  |  |
| **Age** |  |  |  |  |
| < 70 years old | 3.23 | 0.659 | 5.43 | 0.545 |
| ≥ 70 years old | 4.40 |  | 10.80 |  |
| **Performance status** |  |  |  |  |
| 0-1 | 4.70 | 0.140 | 9.60 | **0.027** |
| 2 | 2.37 |  | 3.17 |  |
| **BMI^a^** |  |  |  |  |
| <25 kg/m^2^ | 2.33 | **0.009** | 3.77 | 0.106 |
| ≥25 kg/m^2^ | 4.93 |  | 9.60 |  |
| **Histology** |  |  |  |  |
| Squamous | 4.00 | 0.628 | 6.37 | 0.847 |
| Non squamous | 2.37 |  | 9.43 |  |
| **Disease burden^b^** |  |  |  |  |
| High | 1.77 | **0.008** | 9.60 | 0.139 |
| Low | 4.67 |  | 4.83 |  |
| **Brain metastases** |  |  |  |  |
| Yes | 1.63 | 0.506 | 4.80 | 0.953 |
| No | 3.97 |  | 9.47 |  |
| **Liver metastases** |  |  |  |  |
| Yes | 1.73 | **0.002** | 4.83 | 0.238 |
| No | 4.80 |  | 9.47 |  |
| **Bone metastases** |  |  |  |  |
| Yes | 2.10 | **0.024** | 3.77 | **0.011** |
| No | 4.80 |  | 10.33 |  |
| **LN^c^ metastases** |  |  |  |  |
| Yes | 4.67 | 0.607 | 9.47 | 0.758 |
| No | 2.37 |  | 6.77 |  |
| **Baseline albumin levels** |  |  |  |  |
| < 3.5 g/dl | 1.70 | **0.005** | 1.70 | **0.003** |
| ≥ 3.5 g/dl | 4.40 |  | 9.57 |  |
| **Baseline LDH^d^ levels** |  |  |  |  |
| ≤ 247 units/liter | 3.97 | 0.078 | 9.90 | **0.040** |
| > 247 units/liter | 1.53 |  | 3.70 |  |
| **NLR^e^** |  |  |  |  |
| ≤ 3 | 4.93 | **0.024** | 9.60 | 0.139 |
| > 3 | 2.53 |  | 5.27 |  |
| **Steroid administration^f^ for supportive reasons** |  |  |  |  |
| Yes | 1.27 | **0.013** | 2.53 | 0.051 |
| No | 4.70 |  | 9.60 |  |
| **ATB^g^ administration** |  |  |  |  |
| Yes | 1.97 | 0.062 | 4.00 | 0.301 |
| No | 4.80 |  | 9.47 |  |
| **Prolonged ATB administration** |  |  |  |  |
| Yes | 1.57 | **<0.001** | 2.53 | **0.001** |
| No | 4.93 |  | 9.90 |  |
| **PPis^h^ administration** |  |  |  |  |
| Yes | 2.33 | 0.119 | 6.37 | 0.099 |
| No | 4.70 |  | 9.47 |  |
| **Inhalational steroid administration** |  |  |  |  |
| Yes | 2.10 | 0.467 | 4.00 | 0.558 |
| No | 4.00 |  | 9.43 |  |

a: BMI=Body mass index, b: High disease burden: > 2 organs with metastatic disease, low disease burden: ≤ 2 organs with metastatic disease, c: LN=Lymph Nodes, d: LDH=Lactate dehydrogenase, e: NLR=neutrophil/lymphocyte ratio, f: Administration of > 10 mg of prednisolone equivalent for ≥ 10 days, g: ATB=Antibiotics, h: PPis=Proton pump inhibitors
