# Supplementary material for: Prediction of outcome in patients with non-small cell lung cancer treated with second line PD-1/PDL-1 inhibitors based on clinical parameters: Results from a prospective, single institution study
Source: PLoS One. 2021 Jun 1;16(6):e0252537. doi: 10.1371/journal.pone.0252537 (PMC8168865; doi:10.1371/journal.pone.0252537)

S1 Fig: Mann-Whitney U test examining the effect of the duration of ATB administration in days on DS rates. On the left side are the days on ATB of the patients that experienced PD and on the right side the days on ATB of those who had DS (PR or SD) as response to ICI administration.


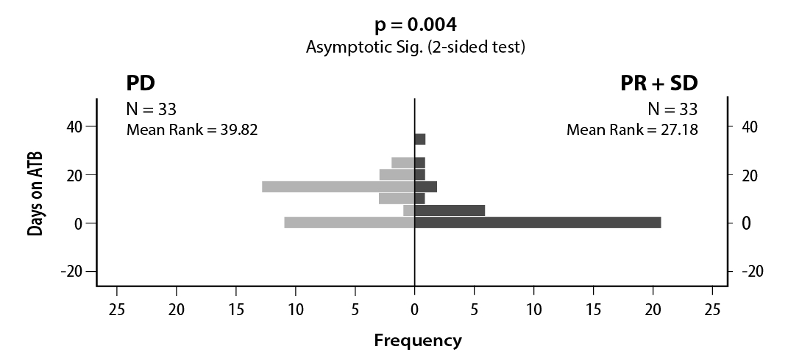

Supplement: S1 Fig — On the left side are the days on ATB of the patients that experienced PD and on the right side the days on ATB of those who had DS (PR or SD) as response to ICI administration. (DOC) [file pone.0252537.s007.doc]
