# Supplementary figures and images for: Prediction of outcome in patients with non-small cell lung cancer treated with second line PD-1/PDL-1 inhibitors based on clinical parameters: Results from a prospective, single institution study
Source: PLoS One. 2021 Jun 1;16(6):e0252537. doi: 10.1371/journal.pone.0252537 (PMC8168865; doi:10.1371/journal.pone.0252537)

S2 Fig: Kaplan-Meier curves on the effect of ATB administration on PFS (A) and OS (B).


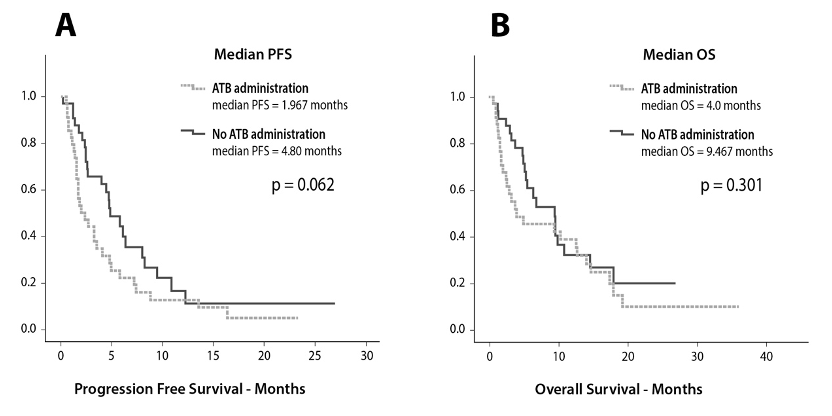

Supplement: S2 Fig — (DOC) [file pone.0252537.s008.doc]

S3 Fig: Kaplan-Meier curves on the effect of steroid administration > 10 mg on PFS (A) and OS (B).


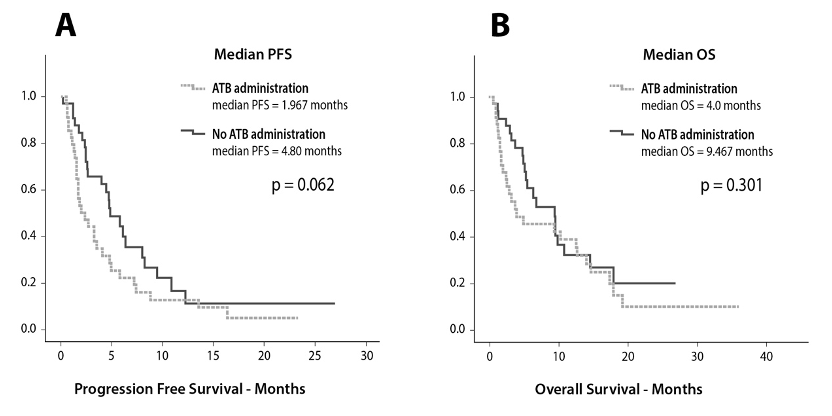

Supplement: S3 Fig — (DOC) [file pone.0252537.s009.doc]

S4 Fig: Kaplan-Meier curves on the effect of chronic PPis administration on (A) PFS and (B) OS.


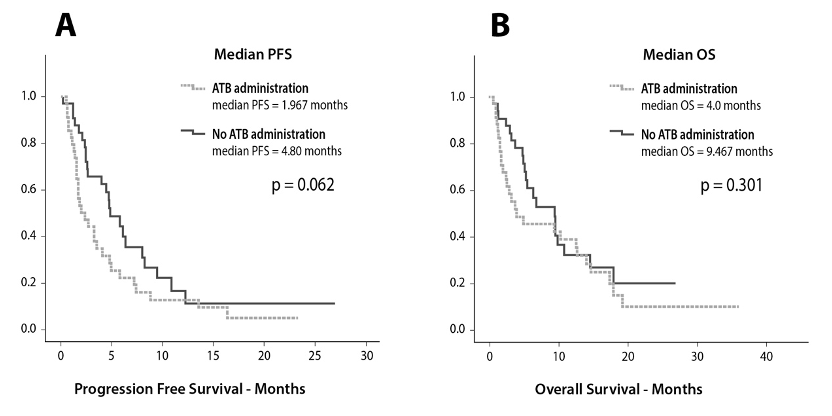

Supplement: S4 Fig — (DOC) [file pone.0252537.s010.doc]
