# Supplementary material for: Prediction of outcome in patients with non-small cell lung cancer treated with second line PD-1/PDL-1 inhibitors based on clinical parameters: Results from a prospective, single institution study
Source: PLoS One. 2021 Jun 1;16(6):e0252537. doi: 10.1371/journal.pone.0252537 (PMC8168865; doi:10.1371/journal.pone.0252537)

**S5 Fig: Kaplan-Meier curves on the effect of inhalational steroids administration on (A) PFS and (B) OS.**


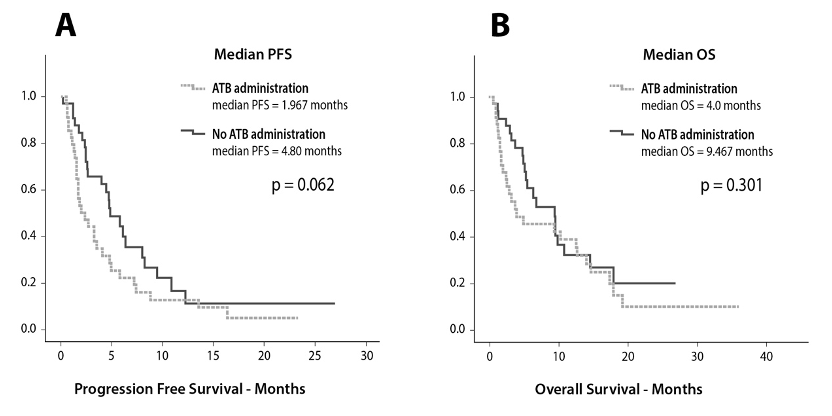

Supplement: S5 Fig — (DOC) [file pone.0252537.s011.doc]
