# Supplementary material for: Prediction of outcome in patients with non-small cell lung cancer treated with second line PD-1/PDL-1 inhibitors based on clinical parameters: Results from a prospective, single institution study
Source: PLoS One. 2021 Jun 1;16(6):e0252537. doi: 10.1371/journal.pone.0252537 (PMC8168865; doi:10.1371/journal.pone.0252537)

S6 Fig. Kaplan Meier curves depicting the effect of the following parameters on OS (A) Baseline albumin levels <3.5 g/dl (B) Presence of bone metastases


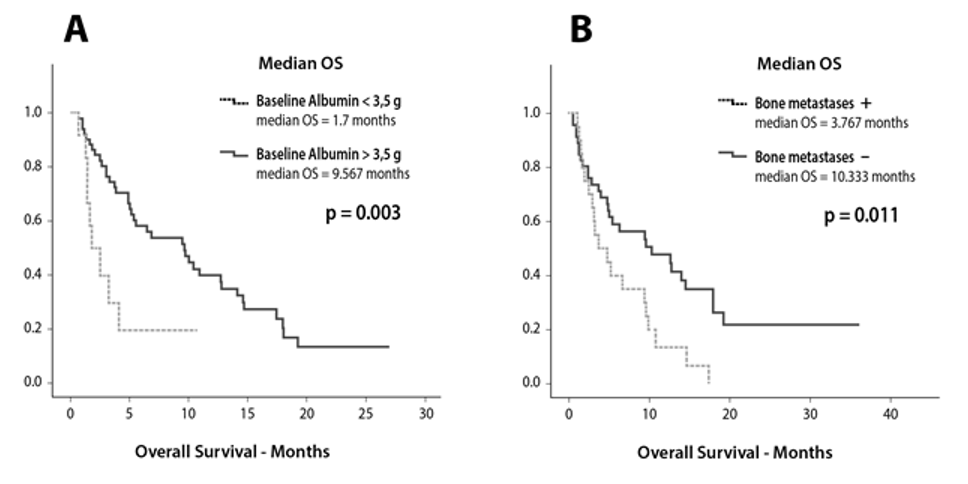

Supplement: S6 Fig — (DOC) [file pone.0252537.s012.doc]
